# Speciality Grand Challenge: Existing and emerging issues for physical activity in the prevention and management of disease and the promotion of wellbeing

**DOI:** 10.3389/fspor.2025.1570340

**Published:** 2025-02-21

**Authors:** David Robert Broom

**Affiliations:** Centre for Physical Activity, Sport and Exercise Sciences, Coventry University, Coventry, United Kingdom

**Keywords:** exercise, physical activity, research development, sedentary, wellbeing

## Introduction and context

It is a real privilege to have been appointed as the new editor of the Physical Activity in the Prevention and Management of Disease section for Frontiers in Sports and Active Living. I have big shoes to fill taking over from esteemed Professor Jason Gill, who for awareness taught me as an undergraduate student and is one of many great mentors who instilled my passion for the topic. Whilst this speciality grand challenge article is by no means meant to be an autobiography, I will reflect on personal experiences and knowledge gleaned from being an undergraduate student in the late 90's as well as draw on the present day to day workings of a Professor to illustrate points. In doing so, I hope the issues raised and this approach will resonate with both seasoned academics as well as students who are the physical activity and health researchers and seasoned academics of the future.

It would be remiss of me not to mention that I have written similar pieces with eminent Professors, notably Smith et al. (1) as I reflect once again on the current and emerging issues in the physical activity and health field and also suggest works that would be welcome submissions to the section as follows:

## We study exercise physiology but sedentary physiology is also important

In Smith et al. (1) we make the point that regardless of the institution, Kinesiology, Physical Activity, Sport and Exercise Science students will typically study ‘Exercise Physiology’ each year of their programme as I did for the 3 years of my undergraduate degree. This discipline seeks to understand the acute and chronic effects of exercise on the muscular, cardiovascular, and neurohumoral systems that leads to changes in functional capacity. Exercise physiology also seeks to understand the effects of exercise on pathology and the mechanisms by which exercise affects the risk and treatment of non-communicable diseases. Notwithstanding there is vast evidence that physical activity and exercise improves health and wellbeing. However, it is becoming clear that sedentary behaviour also influences the aforementioned.

Sedentary behaviour has been defined by Tremblay et al. (2) as ‘any waking behavior characterized by an energy expenditure ≤1.5 metabolic equivalents (METs), while in a sitting, reclining or lying posture’. Many early exercise physiology and physical activity epidemiology papers wrongly referred to people as being ’sedentary’ without measuring said specific behaviour. They were likely ‘inactive’ i.e., not meeting physical activity guidelines for health.

There has been a substantial rise in published studies examining sedentary behaviour in the last two decades with evidence highlighting that ‘prolonged’ and ‘excessive’ sedentary behaviour is detrimental to health. It is therefore fundamental that students learn about and study sedentary physiology and with an expanding evidence base, learned institutions should have taught modules specifically on sedentary behaviour.

Whilst many public health-related organisations and government departments have published guidelines on sedentary behaviour, messaging has focused on ‘reducing’ sedentary behaviour with many failing to identify how long is ‘prolonged’ or ‘excessive’ and if specific patterns of sedentary behaviour need to be avoided. Rresearchers are therefore encouraged to maintain the momentum of sedentary behaviour research but focus efforts on achieving a better understanding of quantified guidance for amounts of sedentary behaviour, especially in the context of varying physical activity behaviour. Movement models and guidelines for a 24 h period do exist [Chastin et al. (3) and Dempsey et al. (4)] and more research is needed on the benefits of low intensity physical activity.

## Children are not little adults so older adults must also be considered heterogenous—the importance of geriatric exercise science

Another mainstay of any physical education, sport or exercise science related degree is studying paediatric exercise science. I remember vividly the message from my lecturers that ‘children are not little adults’ which was based partly on work published by the ACSM (5). As such physical activity interventions and training programmes need to be fit for purpose and adapted accordingly for children and young people. My own research works and interests has demonstrated that the same holds true for those in the later years which has arguably not received as much attention or funding as those in the younger years and particularly in the oldest old.

Research into ageing and advances in medicine has led to longer life expectancy. Whilst this is good news, it presents two challenges in that firstly, how do we ensure successful ageing into ‘extreme’ old age? As an aside, I have used the term ’successful’ and not ‘healthy’ ageing which I personally dislike of as rarely do we age healthily or free from disease. ’Successful ageing’ I define as coping with disease and morbidity, maintaining activities of daily living, maintaining social networks, living independently and enjoying having a high quality of life. Secondly, what are people going to do with this extra time and how do we ensure they make physically active choices to ensure successful ageing?

Please forgive me for presenting the potentially perceived sexist language used in my favourite quote by George Bernard Shaw i.e., ‘Man does not cease to play because he grows old. He grows old because he ceases to play’. This speaks to the importance of making physical activity fun and the importance of play. Researchers are encouraged to examine this amongst the oldest old due to the heterogeneity of older adults and how we can ensure that play, physical activity, exercise and sport leads to successful ageing which has significant benefits to individuals and society notwithstanding a reduction in healthcare costs.

## Prevention is better than cure, but we need whole systems approaches to tackle physical inactivity

Residing in England, I was very pleased to see that prevention is now at the core of the new governments pledge on the National Health Servicev (NHS), with a clear case being made for shifting to a more proactive and preventative healthcare system through community-based interventions (6). Physical activity has a clear role to play in the preventative agenda, as it has long been recognised as an established behavioural determinant of population health and significant in both the prevention and management of numerous health conditions. But getting people to be active is challenging and despite the efforts of many organisations including the World Health Organization (7) many global data sets have seen a plateau or only small increases in physical activity behaviour.

Shearn et al. (8) state that physical activity behaviour is complex. People's activity status, like with many aspects of life, are influenced by a combination of things including personal, social and environmental factors. It is not easy to unravel the many reasons why some people are more active than others as some of these reasons are personal, due to preferences and motivation. Some are due to circumstances, such as family or work commitments sapping time and energy and in some places is it is easier to be active than others due to the environment e.g., where walking routes and cycle paths are safe, and facilities accessible. All of these influences interact to produce the patterns of physical activity that we actually see in different places over time.

Recognising this complexity means that when trying to understand how a programme or intervention works or doesn't work well in getting more people to be physically active, it is important to consider the range of contextual factors e.g., the whole system, it is operating within. A whole systems approach is defined as ‘responding to complexity’ through a ‘dynamic way of working’, bringing stakeholders, including communities, together to develop ‘a shared understanding of the challenge’ and integrate action to bring about sustainable, long-term systems change (9).

Ding and Ukelund (10) highlight that the physical activity and health field has flourished in the last 70 years and research is clear that physical activity benefits physical, psychological, social and functional health. Whilst there is still a necessity for such works to optimise programmes and interventions, researchers are encouraged to focus on research that addresses the ‘how’ do we get people more physically active using whole systems and place-based approaches. How can we use innovative approaches to evidence about what creates a space for people to learn and then to act on them? How do we build relationships with key people and policy makers and how do we make sense of the impact of this work?

## The need for an appropriately qualified and experienced workforce to treat and mitigate physical inactivity related diseases and improve health and wellbeing

As well as my clear enthusiasm for prevention as aforementioned, the reality is that many of the world's population are living with long-term conditions stressing the importance of treatment. Valabhji et al. (11) calculated that the overall prevalence of people living with multiple long-term conditions (multi morbidity) in England is 14.8% but this ranged from 0.9% in those aged ≤19 years to 68.2% in those aged ≥80 years. Supporting my earlier point regarding healthy ageing being an inappropriate term for the masses.

For many conditions, exercise therapy may be as effective as pharmacological therapy (12) and physical activity interventions are highly cost effective. For example, the NHS “Long Term Plan” (13) advocated exercise programmes for patients with cardiovascular disease (CVD) to prevent 14,000 premature deaths and (what was formerly) Public Health England acknowledged that embedding physical activity into clinical care pathways in acute settings was required (14). However, we need an appropriately qualified and experienced workforce to treat and mitigate physical inactivity related diseases and at this moment in time it is debateable whether we have this.

Clinical Exercise Physiologists (CEPs) specialise in exercise testing and assessment, alongside the design, delivery and evaluation of evidence-based exercise interventions. A CEPs scope of practice encompasses apparently healthy individuals to those with chronic and complex conditions, along the care pathway from primary prevention, through acute management, to rehabilitation and maintenance. They design and deliver Interventions that are exercise or physical activity-based and also include health and physical activity education, advice and support for lifestyle modification and behaviour change. CEPs work in a range of primary, secondary and tertiary care settings as part of a multidisciplinary team of health care and rehabilitation providers and in community settings.

I am a founding member of Clinical Exercise Physiology—UK (see https://www.clinicalexercisephysiology.org.uk) which has been established to ensure CEPs get the same recognition as other Allied Health Professionals. I hope to see the day when CEPs are an integral part of all multidisciplinary teams of clinicians and allied health professionals working across primary, secondary, tertiary and community-based services in the public, private or charitable sectors with plenty of job opportunities for Sports and Active Living graduates. Research that highlights the need, demand and benefits of an appropriate workforce to prevent and treat disease through physical activity are essential for future change.

## The replication crisis must be addressed

There is constant pressure on researchers to deliver high quality, innovative and novel work to meet the demands of notable funders as well as assessments of the quality of research such as the Research Excellence Framework (15). Working at a higher education institution in the UK, ‘REF 2029’ is a guaranteed agenda item of every senior leadership team meeting I attend. However, whilst I see the need for research assessment exercises such as REF, in my opinion they lead to what Smith et al. (1) refer to as a ‘replication crisis’**.**

Ritchie (16) provides compelling evidence that in a number of scientific disciplines not enough research is being replicated. He asserts that ‘*hardly anyone runs replication studies…in economics, a miserable 0.1% of all articles published were attempted replications of prior results*’ (page 34).

To address this issue in Sports and Active Living research, it is suggested that the discipline identifies if it has a replication crisis (which personally I think it does!). We should recognise and support the value of replication studies through funding and publication mechanisms and therefore researchers are encouraged to work in interdisciplinary teams to submit replication studies to the section. However, there must be a clear focus on any differences and variation in the results from any original publications, the key reasons for these and the practical application.

## A call for action

As new section editor I welcome all high-quality submissions that examines physical activity in the prevention and management of disease that answers appropriate research questions. However, I particularly welcome submissions and research that:

• Enable us to define excessive sedentary behaviour and patterns that are harmful to health.
• Examine physical activity behaviour in the oldest old.
• Test and evaluate whole-systems and place-based approaches to reduce physical inactivity.
• Explore the role of CEPs operating in multi-disciplinary teams.
• Combats the replication crisis.

## References

[1] Smith A Broom D Murphy M Biddle S. A manifesto for exercise science—a vision for improving the health of the public and planet. J Sports Sci. (2022) 40(10):1110–5. 10.1080/02640414.2022.2049083 35262464

[2] Tremblay MS Aubert S Barnes JD Saunders TJ Carson V Sedentary behavior research network (SBRN)—terminology consensus project process and outcome. Int J Behav Nutr and PA. (2017) 14(75). 10.1186/s12966-017-0525-8 PMC5466781 28599680

[3] Chastin SFM McGregor DE Biddle SJH Cardon G Chaput JP Striking the right balance: evidence to inform combined physical activity and sedentary behavior recommendations. J Phys Acti Health. (2021) 18(6):631–7. 10.1123/jpah.2020-0635 33990471

[4] Dempsey PC Biddle SJH Buman MP Chastin S Ekelund U New global guidelines on sedentary behaviour and health for adults: broadening the behavioural targets. Int J Behav Nutr Phys Act. (2020) 17(1):151. 10.1186/s12966-020-01044-0 33239026 PMC7691115

[5] American College of Sports Medicine. Children aren't miniature adults: similarities and differences in physiological responses to exercise part 1. ACSMs Health Fit J. (2021) 5(5):11–7.

[6] NHS Confederation and Carnal Farrar. Paving a new Pathway to Prevention. London: NHS Confederation and Carnal Farrar (2024). Available online at: www.carnallfarrar.com/wp-content/uploads/2024/10/Pathway-to-prevention-CF.pdf

[7] World Health Organization. Global Action Plan on Physical Activity 2018–2030: More Active People for a Healthier World. Geneva: World Health Organization (2018). Available online at: https://www.who.int/publications/i/item/9789241514187

[8] Shearn K Vincent R Harris K Davies R. Rationale for Whole Systems Approaches and What it Means for Evaluation and Learning. London: Sport England (2024). Available online at: https://evaluatingcomplexity.org/resources/new-articles

[9] Buck D Baylis A Dougall D Robertson R. A Vision for Population Health: Towards a Healthier Future. London: The King’s Fund (2018). Available online at: www.kingsfund.org.uk/publications/vision-population-health

[10] Ding D Ekelund U. From London buses to activity trackers: a reflection of 70 years of physical activity research. J Sport Health Sci. (2024) 13(6):736–8. 10.1016/j.jshs.2024.06.001 38851584 PMC11336341

[11] Valabhji J Barron E Pratt A Prevalence of multiple long-term conditions (multimorbidity) in England: a whole population study of over 60 million people. J R Soc Med. (2024) 117(3):104–17. 10.1177/01410768231206033 37905525 PMC11046366

[12] Pedersen BK Saltin B. Exercise as medicine—evidence for prescribing exercise as therapy in 26 different chronic diseases. Scand J Med Sci Sports. (2015) 25(Sup 3):1–72. 10.1111/sms.12581 26606383

[13] NHS. The NHS Long Term Plan. London: NHS (2019). Available online at: www.longtermplan.nhs.uk

[14] England P Active E. every day: An Evidence-based approach to Physical Activity. London: Public Health England (2024).

[15] Research Excellence Framework (REF). (2025). Available online at: www.ukri.org/who-we-are/research-england/research-excellence/research-excellence-framework/

[16] Ritchie S. Science Fictions: Exposing Fraud, Bias, Negligence and Hype in Science. London: The Bodley Head (2020).

